# The Nutritional Potential of the Native Australian Green Plum (*Buchanania obovata*) Compared to Other Anacardiaceae Fruit and Nuts

**DOI:** 10.3389/fnut.2020.600215

**Published:** 2020-12-16

**Authors:** Selina Fyfe, Heather E. Smyth, Horst Joachim Schirra, Michael Rychlik, Yasmina Sultanbawa

**Affiliations:** ^1^Queensland Alliance for Agriculture and Food Innovation, The University of Queensland, Coopers Plains, QLD, Australia; ^2^Centre for Advanced Imaging, The University of Queensland, Brisbane, QLD, Australia; ^3^Chair of Analytical Food Chemistry, Technical University of Munich, Freising, Germany

**Keywords:** Anacardiaceae, green plum, *Buchanania obovata*, nutrition, mango, cashew, pistachio, family

## Abstract

The native Australian green plum (*Buchanania obovata*) is a small fruit that grows in the northern parts of the Northern Territory and Western Australia. The fruit belongs to the family Anacardiaceae, which includes the other agriculturally important fruit mangoes, pistachios and cashew nuts. The green plum is a favored species of fruit for the Aboriginal communities and an important bush food in the Northern Territory. To date, only minimal scientific studies have been performed on the green plum as a food. This review is about plant foods in the family Anacardiaceae and the key nutritional compounds that occur in these fruit and nuts. It looks at the more traditional nutrient profiles, some key health metabolites, allergens and anti-nutrients that occur, and the role these foods play in the health of populations. This provides a guide for future studies of the green plum to show what nutritional and anti-nutritional properties and compounds should be analyzed and if there are areas where future studies should focus. This review includes an update on studies and analysis of the green plum and how its nutritional properties give it potential as a food for diet diversification in Australia.

## Introduction

The family Anacardiaceae is a member of the flowering plant order Sapindales and contains about 80 genera. There are ~870 species in the family characterized as deciduous or evergreen trees, shrubs and woody vines which contain resin ducts in the bark and that exude resins and gums (1). The fruits of this family are drupes that are fleshy (1). The Anacardiaceae family contains a number of plants that produce foods, some are globally important economically and others are important in smaller communities. They provide nutritional properties and diet diversification to people all throughout the world. The family includes one of the most well-known fruit in the world, the mango (*Mangifera indica*), and the equally well-known cashew nut (*Anacardium occidentale*) and pistachio nut (*Pistacia vera)*. Other foods in the family that are not globally available but are important in the countries they grow in include the fruit of *Pistacia lentiscus*, the marula fruit (*Sclerocarya birrea*), the sumac of the genus Rhus and in particular *Rhus corriaria*, the yellow mombin (*Spondias mombin*), and the chironji (*Buchanania lanzan*).

**Figure 1:**
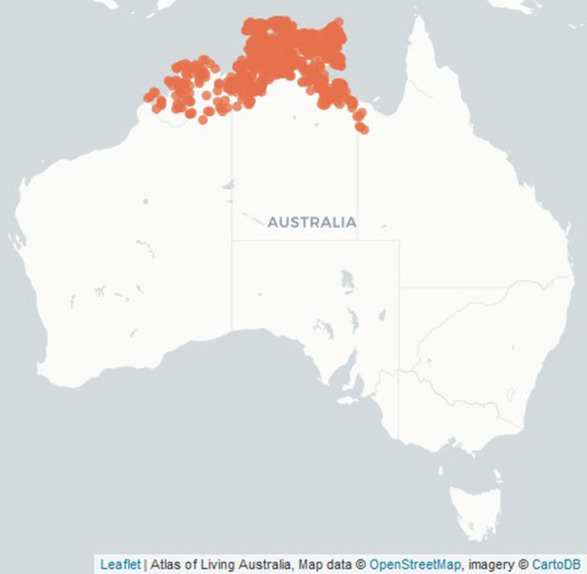
Location of botanically identified *Buchanania obovata* trees in Australia (Atlas of Living Australia).

**Figure 2:**
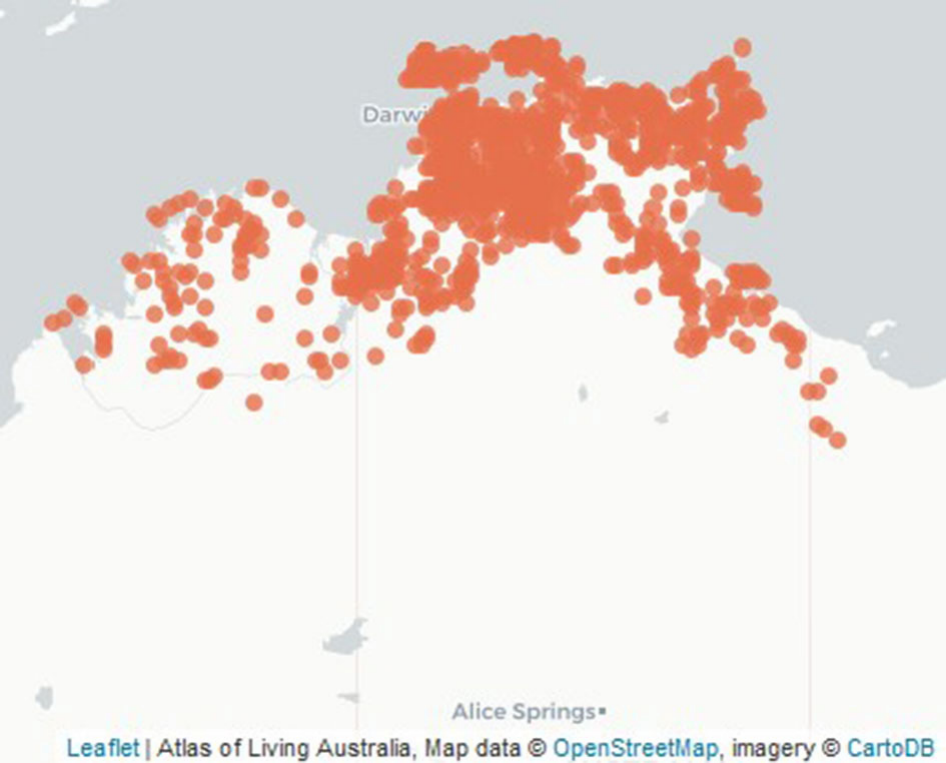
Location of *Buchanania obovata* trees botanically identified in the northern Western Australia and Northern Territory (Atlas of Living Australia).

A less well-known fruit of the family Anacardiaceae is the green plum, the fruit of the tree *Buchanania obovata*. It is a small green/yellow fruit that grows as a drupe and it is a favored species of fruit and an important bush food for Aboriginal people in the Northern Territory and Western Australia where it grows (Figures 1, 2). They are eaten raw from the tree and the individual fruit are also eaten dried or reconstituted (2). Green plums were prepared and stored by Aboriginal people so they could be eaten at a later time, the fruit and seed were pounded into a pulp or paste and sun-dried then stored in sheets of paperbark (2, 3). The accepted name of the plant that grows the green plum fruit is *Buchanania obovata* Engl. (4), it was first described in 1883 (5) and the plant taxonomy is shown in Table 1.

**Table 1:** Taxonomy of *Buchanania obovata* (6).

**Class**	**Green plum classification**
Kingdom	Plantae
Phylum	Charophyta
Class	Equisetopsida
Subclass	Magnoliidae
Superorder	Rosanae
Order	Sapindales
Family	Anacardiaceae
Genus	*Buchanania*
Species	*Buchanania obovata*

There are two published studies on the green plum as a food, however, both of these studies were done on underripe green plums. One study looked at the nutritional properties of the green plum flesh and seed as both parts are eaten. It found the flesh was high in protein [12.8 g/100 g dry weight (DW)], potassium (2274.7 mg/100 g DW) and was a good source of magnesium, calcium and phosphorous. The seed was found to be high in iron (8.15 mg/100 g DW). Both the flesh and seed were found to be high in dietary fiber. There were also high levels of folate found, with the flesh containing 752.4 μg/100 g DW as pterolmonoglutamic acid equivalents (7).

The second study was on the functional properties and phytochemicals of the same underripe green plums. This study found that the flesh had antimicrobial activity against Gram-negative *Escherichia coli* 9001—NCTC and Gram positive *Staphylococcus aurerus* 6571—NCTC bacteria and caused cell wall disintegration and cytoplasmic leakage. Antioxidant testing using the 2,2-diphenyl-1-picrylhydrazyl (DPPH) assay showed the flesh had high radical scavenging activity (106.3 μM Trolox equivalent/g DW in methanol). Extract of the seed had a pink color and contains a delphinidin-based anthocyanin. Polyphenols found in the flesh and seed include gallic acid, ellagic acid, p-coumaric acid, quercetin, kaempferol, and trans-ferulic acid (8).

These preliminary results show that the green plum has promising nutritional properties and should be further studied, especially when it is at its ripe and edible stage. This review aims to understand how best to study the green plum as a food by analyzing the nutritional properties and importance of other foods from the Anacardiaceae family. It does this by looking at how underutilized plant food can be used as nutrition. Then it looks at foods that are produced by plants in the family Anacardiaceae and how they are used as foods. Then the key nutritional properties of these foods are tabled and reviewed. Next, the known and potential roles of these foods as functional foods and nutraceuticals is reviewed. The properties that could prevent these foods from playing a role in nutrition is looked at, especially the allergenic causing compounds. Then the role that these foods play in nutrition of populations is described, in doing this the role of these foods in nutrition for individuals and populations are both reviewed. Finally, it concludes with how the nutritional properties of foods in the Anacardiaceae family can give insight and understanding for studying the green plum and discovering its nutritional potential.

## Underutilized Plant Foods as Nutrition

Foods are fundamental to the existence of humans and the nutritional value of them has a direct impact on the health of populations (9). Therefore, nutritional components are important to study so the health benefit of these foods can be understood. Fruit and vegetables, or plant foods, are an important part of a healthy diet because they provide valuable carbohydrates, dietary fiber, vitamins, minerals, and trace elements.

Malnutrition can be caused by overnutrition, undernutrition and micronutrient deficiencies. It is estimated that over 2 billion people in the world suffer from micronutrient deficiencies from insufficient intake of vitamins and minerals in the diet (10). There are an estimated 821 million people who are undernourished (11). Malnutrition from food insecurity can be due to insufficient quantity of food, insufficient quality of food and insufficient variety of foods in the diet (11). Across the world 60% of dietary calories comes from the food staples rice, wheat, maize, potato, and soybean, and 90% of dietary calories comes from just 103 of the 30,000 plant species that are edible (12). Adding more variety of plant foods into the diet can increase the micronutrient intake and increase nutrition and health of consumers. Understanding the nutrition composition of Australian native fruit is important to be able to promote them as healthy foods and to add variety to the diets of Indigenous and non-Indigenous people across Australia. There is the possibility of using native Australian fruit for diet diversification in Indigenous and non-Indigenous Australians, to increase nutrition intake and health (7).

At least 37 native plant foods are available commercially in Australia, including 13 fruit. There are many plant nurseries selling bush food plants that can be bought and grown for food. A few of these foods have been studied in detail, but most have had very little modern scientific analysis. The native Australian fruit that has been studied the most intensely is the Kakadu plum from the tree *Terminalia ferdinandiana* which grows in the Northern Territory and Western Australia. The Kakadu plum has extremely high levels of vitamin C with up to 32% of dry weight (322.2 ± 2.1 mg/g DW) being recorded (13). Even the kernels of the Kakadu plum can be used for their nutritional value due to their content of dietary fiber (21.2% DW), energy (2,065 kJ/100 g DW), potassium (6,693 mg/kg DW), calcium (5,385 mg/kg DW), zinc (60 mg/kg), and iron (61 mg/kg DW). The kernels also contain high levels of protein (32.0% DW) and fat (35.1% DW) and contain linoleic, palmitic, and oleic acids (14). In Australia, native fruit that have been traditionally eaten by Indigenous Australians are allowed to be commercialized as they are classed by the Food Standards Australia New Zealand (FSANZ) as traditional foods with a history of consumption in Australia and are not considered novel (15).

## Foods of the Family Anacardiaceae

The tree *Mangifera indica* is an evergreen tree that produces the major fruit crop mango (16). Many different mango cultivars are eaten and they vary in size, shape, color, flavor, and fiber quantity (16). Mangoes can be oval, round, heart-shaped, kidney-shaped, or slender and long, can be as small as plums or weigh up to 2.3 kg and can vary in color between red, yellow, and green (17). Mango is eaten as a fresh fruit with the pulp and occasionally the skin consumed. Processed mango food products are made from the flesh and consumed throughout the world including juice beverages, candies and fruit bars, jams, jellies, pickles, and powder mixes (18).

The cashew tree (*Anacardium occidentale*) grows the cashew apple and cashew nut that are eaten across the world. The kernel from the seed is known and eaten as the cashew nut and the swollen pedicel of the flower stalk is eaten as fresh cashew apple and as a juice (19). The tree is believed to be native to Brazil and was moved to other parts of the world in the sixteenth century by the Portuguese (19). The apples are edible but are often not used or eaten, in Northeast Brazil about 90% of the apple is left in the field during harvest (20). The kernel is mostly consumed roasted and salted and is used in the food industry, particularly in chocolate, pastry, and biscuit factories. The oil from the kernel is used in food and cosmetic products (21). The juice from the apple can be extracted and used to produce vinegar, syrup, and alcohol (21) or can be fermented and used to produce added value products, such as lactic acid, dextran, mannitol, and oligosaccharides (20). The shell and peel can be used as fuel for the drying and cooking processes of the nut and the peel is used as food for livestock. The oil from the cashew nut shell is extracted and purified and used in the manufacturing of chemical products (21).

The pistachio nut grows on the tree *Pistacia vera* and is actually a kernel that grows inside the seed pod and flesh of the fruit. The pistachio tree is thought to be indigenous to Iran and is grown in California in the US, and across from Afghanistan to the Mediterranean region (22). The kernel is often eaten fresh or roasted, sometimes with salt or flavoring, they are used in sweets and desserts and for their yellow-green color (22, 23). The shell around the pistachio kernel splits naturally before it is harvested which allows it to be sold in-shell (23).

In the same genus as the pistachio is *Pistacia lentiscus*, the mastic or lentisk tree, which grows in the Mediterannean basin and produces the lentisc fruit with an edible oil that is used in the daily diet of Tunisians (24). In Tunisia it is used in salads and pastries and served as a condiment, there is interest in furthering its use as a vegetable oil (24). Another food product is collected by incisions made into the side of the tree and the resin that drips out is used as a chewing gum known as mastic gum (25).

The *Sclerocarya birrea* tree grows in Africa and produces marula fruit. It grows in the African triangle from Cape Verde, to the Horn of Africa, to the Cape of Good Hope (26). Marula is a pale yellow fruit and has a juicy mucilageinous flesh that is eaten fresh or is fermented to make beer and other alcoholic beverages (27, 28). The flesh is tart, sweet, and refreshing with a slight turpentine aroma (28). The flesh of the fruit is used for making the South African liquor Amarula Cream (28). The seed is opened to obtain and eat the macadamia-like kernel inside (26, 29).

The name of the fruit sumac is used for about 35 species in the genus *Rhus*. One of the most common species of sumac is *Rhus corriaria* which is in Turkey, Syria, and throughout the Arab world (30, 31). It is used as a spice, condiment, appetizer, and a souring agent (32).

*Spondias mombin*, whose fruit is known as yellow mombin, grows in the tropical parts of America, Asia, and Africa (33). In Brazil it is harvested wild and its flesh is eaten and used in other food products (33). *Spondias purpurea* are native to Ecuador and the fruit, known as ovo, is sweeter and more aromatic then the yellow mombin and usually eaten fresh or in jams, ice-creams, or beverages (34).

The *Buchanania lanzan* tree produces seeds that are edible and are known as chironji, char, achar, or the cuddapah almond. It occurs in the wild in India where it is eaten and used in cooking and where the tree is used as a medicinal plant (35). The tree is also found in Myanmar, Sri Lanka, Malaysia, Burma, and Nepal (35, 36). To obtain the kernel, the fruit are harvested, the skin allowed to blacken in storage then removed, the seed is washed and dried and then the seed shell is removed from the kernel (37). The kernel of the seed is eaten either raw or roasted, it can be used as a substitute for almonds and is used in manufacturing sweet-meats and confectionery (37–39). Its use as a cooking spice is also starting to become more well-known outside of India. The fruit are a small drupe, growing to 12 mm and they turn from green to reddish to black, the fruit weight is made up of about 16–18% kernel (35, 39).

Like *B. obovata, Buchanania arborescens* is also native to Australia and its fruit have also been eaten by Aboriginal Australians. It also has fruit that are a small globular drupe, growing to 10 mm in diameter. The fruit turn reddish-purple to purple-black when it ripens and its fruiting time is the same as that of the green plum (2). The fruit are eaten raw and taste sweet and pulpy and have a thin rind (40). The tree is known as the little gooseberry tree and the satinwood tree. It grows across the northern parts of the Northern Territory and Queensland in Australia and in East Timor, West Papua, and Papua New Guinea (6).

The Burdekin plum or gambozia (*Pleiogynium timoriense*) is a dark purple fruit with a thin layer of flesh which is sour and astringent when it falls from the tree but becomes palatable a few days after harvesting (41, 42). It grows in Queensland in Australia, and in the area between Queensland, the Philippines, Indonesia, and the Cook Islands (41, 43) and is cultivated as an ornamental plant in Egypt (42). The fruit are eaten by Indigenous Australians and the flesh has been used to make jam (41).

There are some other species in the Anacardiaceae family that are found throughout the world and are used as foods, but which have not have as much in-depth nutritional study. The fruit from *Pistacia palaestina* are used in the Middle East as a component of the “Zaatar” a blend eaten daily with bread, olive oil and tea (44). Likewise with terebinth, *Pistacia terebinthus*, which is an important spice plant in Turkey and is eaten as an appetizer (30). The fruit of the tree *Schinus molle* is known throughout the world as pink pepper and sometimes as Peruvian pepper. It is not related botanically to true black pepper (*Piper nigrum*). Fruit from other *Mangifera* species are used in similar ways to the closely related mango in parts of Asia including *M. caesia, M. foetida*, and *M. parvifolia* (45).

## Nutritional Properties of the Family Anacardiaceae

Nutritional analysis on some of the foods from the Anacardiaceae family has been published. The mango, pistachio nut, and cashew nut have been studied in detail as they are widely eaten foods. Other foods have had some nutritional studies done but not the in-depth analysis of the more widely eaten foods, while others, such as the green plum have had very little. The foods of the Anacardiaceae family provide valuable nutrients and health benefits to the individuals who eat them and add diversity of nutrients into the diet.

To understand the nutritional potential that the green plum might have, the nutrition properties of the foods in the Anacardiaceae are described in detail to see if there are trends or key nutrients that occur in this family. This review first looks at the more traditional nutrients then later in the review looks at phytonutrient compounds in them that effect health and may be able to be used as functional foods or nutraceuticals. The traditional nutrients include the proximate analysis and the key minerals present. Then the fatty acids and lipids are reviewed as this group of foods contains a number of edible kernels. Folates and vitamin C are essential vitamins that are important to health and obtained through diet, and folate was found to be high in the initial study of the green plum (7).

There are large databases of food nutrients that give nutritional profiles of some of these foods. Of these databases, mango, pistachio and cashew can be found in the Australian Food Composition Database (46), the FoodData Central (47), Ciqual the French food composition table (48), and the McCance and Widdowson's composition of foods integrated dataset (49). It was decided that for these three well-studied foods only the USDA data would be included for review to keep the data pertinant.

The nutrients and phytonutrients of mango, pistachio, and cashew have been previously looked at individually in other reviews and book chapters, but not as a plant family together. These reviews include the constituents of cold pressed pistachio oil (50), the bioactive compounds and the functional effects of pistachio green hull (51), cancer preventive and anticancer therapeutic potential of mango and its phytochemicals (52), how the levels of lupeol, mangiferin, and phenolic acids can be regulated and improved in mangoes (53), the potential for the fat fraction of mango kernels to be used as a healthy food ingredients and cocoa butter alternatives (54), the ethnomedical and pharmacological activity of compounds in mango (55), the major polyphenols that are in mango and their potential health benefits (56) the efficacy of cashew nut consumption on lipid profile and blood pressure (57), the effect of cashew nut consumption on lipid profile (58), and the effect of cashew nuts on cardiovascular risk factors and blood pressure (59).

### Proximates and Minerals

The proximate and some mineral components of the Anacardiaceae fruit flesh are in Table 2. The trends seen across the flesh of these fruit are that the mango, marula, Burdekin plum, yellow mombin, and ovo contain large amounts of moisture and carbohydrate. The sumac and terebinth are comparably very low in moisture and much higher in fat. Potassium is the most abundant mineral in all of the fruit flesh, but calcium is found in considerably higher levels in sumac and terebinth then the other fruit.

**Table 2:** Key proximate and mineral levels of fruit flesh of the Anacardiaceae family, results as fresh weight.

	**Green plum**	**Mango**	**Marula**	**Sumac**	**Terebinth**	**Yellow mombin**	**Ovo**	**Burdekin Plum**
Plant or fruit part	Underripe flesh	Flesh	Flesh	Flesh	Flesh	Flesh	Flesh	Flesh
References	(7)	(60)	(61)	(30)	(30)	(33)	(34)	(62)
Moisture g/100 g	79	83.46	86.4	6.0	4.0	83.66	77.6	72.7
Protein g/100 g	2.69	0.82	–	2.3	6.4	1.06	0.7	1.3
Fat g/100 g	0.53	0.38	0.54	17.4	42.4	0.62	0.2	1.3
Fiber g/100 g	11.6	1.6	–	–	–	1.87	0.5	18.4
Carbohydrate g/100 g	4.5	14.98	–	–	–	13.90	19.1	25.0
Potassium mg/100 g	478	168	355	526	762	288	250	458
Iron mg/100 g	0.79	0.16	9.9	18.1	10.7	0.3	0.72	0.9
Phosphorous mg/100 g	46	14	18	–	–	33	42	–
Calcium mg/100 g	89	11	30	133	310	11	17	241
Magnesium mg/100 g	120	10	16	77	97	15	–	32
Zinc mg/100 g	0.5	0.09	5.8	2.6	3.9	–	0.02	1.1

*– means a reported result was not available*.

The results in Table 3 show the trends across the kernels. They do not contain much water, but are very high in fat and in protein. The kernels are also high in potassium and phosphorous, and good sources of calcium and magnesium. The cashew nut kernel is made up mostly of fat, carbohydrate, and protein (Table 2) (60). Analysis of cashew nut kernels from six different parts of India as well as the Ivory Coast, Brazil, Vietnam, Mozambique, and Kenya show consistent protein results with a mean and standard deviation of 21.3 ± 0.8 g/100 g, consistent carbohydrate of 20.5 ± 1.5 g/100 g and consistent energy levels 2,525 ± 35.8 kJ/100 g (64).

**Table 3:** Key proximate and mineral levels of seed and kernels of the Anacardiaceae family, results as fresh weight unless stated.

	**Green Plum**	**Chironji**	**Cashew nut**	**Pistachio**	**Marula**
Plant or fruit part	Underripe seeds	Kernel	Kernel	Kernel	Kernel (dry weight)
Reference	(7)	(63)	(60)	(60)	(29)
Moisture g/100 g	35	3.6	5.2	4.37	–
Protein g/100 g	2.0	43.24	18.22	20.16	36.4
Fat g/100 g	1.2	38	43.85	45.32	47.0
Fiber g/100 g	57.0	18.5	3.3	10.6	–
Carbohydrate g/100 g	4.4	12.96	30.19	27.17	–
Potassium mg/100 g	185	–	660	1025	364
Iron mg/100 g	5.3	4.8	6.68	3.92	2.77
Phosphorous mg/100 g	30	593	593	490	1040
Calcium mg/100 g	30	70	37	105	154
Magnesium mg/100 g	32	275	292	121	421
Zinc mg/100 g	0.24	3.32	5.78	2.2	6.24

*– means a reported result was not available*.

The USDA data (Table 3) shows potassium is the most abundant mineral present in cashew nut kernels followed by phosphorous and magnesium which is consistent with the results of Rico et al. (64) (622, 503, and 249 mg/100 g). The marula kernel has much higher levels of phosphorous than the other kernels and higher levels of magnesium, but it has lower levels of potassium. The high levels of phosphorous in the kernels were confirmed in a study of marula from Kenya which found levels of 782 and 741 mg/100 g DW in the kernel (61). As sources of potassium, calcium, and magnesium, the Anacardiaceae kernels provide important health benefits. Potassium is critical for muscle function and nerve transmission and involved in energy metabolism glycogenesis and cellular growth and division (65). Phosphorous is critically important and is mostly found in bone with some in soft tissue and in the phospholipids of erythrocytes and plasma lipoproteins (65). Magnesium is in bones, soft tissue and in all compartments of cells performing many cellular reactions and involved in at least 300 enzymatic steps in metabolism (65). Analysis of underripe green plums show the flesh has similarly high levels of potassium, calcium, and magnesium to sumac and terebinth. The green plum seed that was analyzed was whole including the seedcoat, thus analysis of the kernel to compare with nutritional properties of other kernels in the Anacardiaceae family could determine if it has a similar profile (7).

### Fatty Acids and Lipids

The kernels and some of the fruit of the Anacardiaceae family have high levels of fat which indicates they are a good source of energy and good for membrane health (66). Table 4 shows the total lipids and fatty acid concentrations in some of the flesh and kernels of Anacardiaceae fruit. It is significant that the kernels and even some of the flesh have high lipid contents. All of them show similar fatty acid profiles, with the highest fatty acid levels of the unsaturated 18:1, then the polyunsaturated 18:2 and the saturated 16:0, then the saturated 18:0 and with low or trace levels of some other fatty acids also present.

**Table 4:** Fatty acid content of Anacardiaceae seeds showing total fatty acid groups and those with highest presence; all in g/100 g fresh weight except for marula kernel and lentisc flesh which are in dry weight.

**Fatty acid**	**Fatty acid** ** common name**	**Mango**	**Lentisc** ** (dry weight)**	**Pink Pepper**	**Sumac**	**Terebinth**	**Cashew** ** Nut**	**Pistachio Nut**	**Chironji**	**Marula (dry weight)**
Fruit part		Flesh	Flesh	Flesh	Flesh	Flesh	Kernel	Kernel	Kernel	Kernel
Reference		(60)	(24)	(67)	(30)	(30)	(60)	(60)	(38)	(29)
Total lipids		0.38	42.54	5.35	17.4	42.4	43.85	45.32	50	47
Total saturated fatty acids	Butyric acid	0.092	10.2	1.55	4.66	10.34	7.783	5.907	19.05	13.78
4:0	Caproic acid	0	–	–	–	–	0	0	–	–
6:0	Caprylic acid	0	–	–	–	–	0	0.012	–	–
8:0	Capric acid	0	–	–	–	–	0.015	0	–	–
10:0	Lauric acid	0	–	–	–	–	0.015	0.004	–	–
12:0	Tridecylic acid	0.001	–	0.004	–	–	0.015	0	–	–
14:0	Pentadecylic acid	0.013	–	0.031	–	–	0.015	0.019	0.1	0.05
16:0	Margaric acid	0.072	9.9	1.196	3.67	9.20	3.916	5.265	15.15	7.35
17:0	Stearic acid	–	–	0.047	–	–	0.046	0.009	–	–
18:0	Arachidic acid	0.004	0.48	0.115	0.82	1.06	3.223	0.478	3.8	5.22
20:0	Behenic acid	–	0.003	0.004	0.12	0.08	0.266	0.046	–	0.59
22:0	Lignoceric acid	–	–	0.039	0.05	0	0.173	0.04	–	0.18
24:0		–	–	0.113	–	–	0.101	0	–	0.39
Total monounsaturated fatty acids	Myristoleic acid	0.14	22.5	1.098	6.71	15.56	23.797	23.257	27.9	30.79
14:1	Palmitoleic acid	–	–	0.012	–	–	0	0	–	–
16:1	Oleic, Vaccenic and Elaidic acid	0.067	0.545	0.107	–	–	0.136	0.495	–	0.11
18:1	Gondoic acid	0.075	22.00	0.954	6.71	15.56	23.523	22.674	27.9	30.12
20:1	Erucic acid	0	0.077	0.007	–	–	0.138	0.089	–	0.25
22:1	Nervonic acid	0	–	0.018	0	0	–	0	–	0.31
Total polyunsaturated fatty acids		0.071	9.4	2.702	4.77	7.55	7.845	14.38	3.05	2.45
18:2	Linoleic acid Linolelaidic acid	0.019	9.3	2.416	4.77	7.55	7.782	14.091	3.05	2.45
18:3	Linolenic acid	0.051	–	0.219	–	–	0.062	0.289	–	–
18:4	Stearidonic acid	0	–	–	–	–	0	0	–	–
20:2		–	–	–	–	–	0	0	–	–
20:3	Dihomo-y-linolenic and Mead acid	–	–	0.041	–	–	0	0	–	–
20:4	Arachidonic acid and Docosatetraenoic acid	0	–	0.027	0	0.13	0	0	–	–
20:5	Eicosapentaenoic acid	0	–	–	–	–	0	0	–	–
22:5		0	–	–	–	–	0	0	–	–
22:6	Cervonic acid	0	–	–	–	–	0	0	–	–
Total trans fatty acids		0	–	–	–	–	–	0	–	–

*– means a result is not available*.

Chemotaxonomic analysis of seed oils from other plant families show similarities in fatty acid profiles within families and differences between families. The principal fatty acids in the family Ribes are 18:2 and 18:3 with lower percent composition of 18:1 and 16:0, the family Boraginaceae have predominantly 18:1, 18:2, and 18:3 unsaturated fatty acids with some 16:0 also present, the family Ranunculaceae predominantly have 18:2, 18:3, and 16:0 with lower levels of 18:1, the family Onagraceae is mostly 18:2 with a small amount of 16:0 and the family Scrophulariaceae are predominantly 18:2 with lower levels of 18:1 and 16:0 and a few members of this family with 18:3 (68). The family Proteaceae which contains the macadamia nut (genus *Macadami*a with four species) has higher levels of 16:1 unsaturated then the other families, as well as 18:1 and lower levels of 16:0, 18:2, and 20:1 (69).

The different fatty acids have different roles in health and well-being, so the seeds and their oils from different plant families give diverse benefits nutritionally. The fatty acid profile common to the family Anacardiaceae has 18:1 oleic acid as the most abundant fatty acid, which is stable to oxidation and able to enhance the activity of antioxidants and antipolymerization agents (70). Higher intakes of oleic acid and limited intakes of saturated fats are believed to have beneficial health effects and may help prevent cardiovascular disease (70). The palmitic acid, 16:0 present in the Anacardiaceae family plays a number of important physiological roles including being a part of normal pulmonary surfactant in the lungs essential for breathing, and is present in membrane phospholipids and adipose triacylglycerols (71).

A fatty acid profile of the green plum could confirm if it contains a similar profile to the other fruit and kernels of the Anacardiaceae family and to understand the energy and nutrition that it provides to the people who eat it.

### Folates

Folate is an important vitamin group for health that is not synthesized by the human body so must be consumed in food or supplements. It is used by the body in the synthesis of adenosine, guanosine, thymidine and in many methylation reactions (72). Folates measured on six varieties of mango from India had total folate ranging from 60 up to 138 μg/100 g FW (73). Five varieties of mango bought in Germany contained total folate between 55.8 and 74.5 μg/100 g and the highest folate type present was 5-CH_3_-H_4_folate (5-methyltetrahydrofolate) (74). These findings show mango is a good source of natural folate vitamers (74). The USDA nutrition reports of foods in the Anacardiaceae family as raw cashew nuts 25 μg/100 g, mango 43 μg/100 g, and pistachio nuts as 51 μg/100 g (47). The Vadu mango is a very small mango and was tested in its unripe form as this is how it is eaten. It has the highest level of folate of the mangos studied at 138 μg/100 g fresh weight (FW) (73). The initial study of underripe green plums gave an even higher total folate content of 161 μg/100 g FW (7). Further analysis on folates of green plums as a ripe fruit would give beneficial nutritional information, and understanding the folate levels as it matures could be of interest as a comparison to the underripe Vadu mango.

### Vitamin C

Cashew apple juice has been found to have high levels of vitamin C at 203.5 mg/100 mL which was more than four times higher than the juices of orange (54.7 mg/100 mL), grape (45.0 mg/100 mL), lemon (33.7 mg/100 mL), mango (30.9 mg/100 mL), and pineapple (14.7 mg/100 mL) (75). When mixed with these other juices it boosts the nutrition quality by increasing the vitamin C content, while the other fruits improved the taste and flavor of the cashew apple juice (75). The USDA reported level of vitamin C in mango flesh at 36.4 mg/100 g (2019). Ovo has a similar level of vitamin C at 49 mg/100 g (34). Marula fruit has been found to have high levels of vitamin C with levels up to 2,118 mg/100 g DW (76).

### Amino Acids

Amino acids play important roles in human health and well-being as substrates for protein synthesis, regulators of enzyme activity and protein turnover and many of them have individual roles in tissue and organ functions (77). The amino acid profiles analyzed from Anacardiaceae foods are in Table 5. All of these foods have glutamic acid as their most abundant amino acid. Glutamic acid is used for protein synthesis, used in muscle, it controls the acid-base balance, scavenges ammonia, is used as a nitrogen donor and for nitrogen transport, is a substrate for hepatic ureagenesis and gluconeogenesis and a fuel for intestinal enterocytes and generation of cytotoxic products in immunocompetent cells (77).

**Table 5:** Amino acid content of foods from the Anacardiaceae family g/100 g fresh weight except for marula kernel which is dry weight.

**Amino acid**	**Mango**	**Cashew nut**	**Pistachio nut**	**Marula (dry weight)**
Fruit part	Flesh	Kernel	Kernel	Kernel
Reference	(60)	(60)	(60)	(29)
Alanine	0.082	0.84	0.97	0.90
Arginine	0.031	2.12	2.13	5.23
Aspartic acid	0.068	1.80	1.88	4.62
Cystine	–	0.39	0.29	0.89
Glutamic acid	0.096	4.51	4.3	10.97
Glycine	0.034	0.94	1.01	1.29
Histidine	0.019	0.46	0.51	0.92
Isoleucine	0.029	0.79	0.92	1.20
Leucine	0.05	1.47	1.60	1.74
Lysine	0.066	0.93	1.14	0.73
Methionine	0.008	0.36	0.36	0.59
Phenylalanine	0.027	0.95	1.09	1.32
Proline	0.029	0.81	0.94	1.00
Serine	0.035	1.08	1.28	1.49
Threonine	0.031	0.69	0.68	0.87
Tryptophan	0.013	0.29	0.25	0.54
Tyrosine	0.016	0.51	0.51	0.74
Valine	0.042	1.09	1.25	1.43

*– means a reported result is not available*.

The other amino acids most abundant in the kernels are arginine, aspartic acid, glycine, leucine, serine and valine. Arginine is a precursor for urea and nitric oxide synthesis, aspartic acid is a nitrogen donor and transfers it to urea, glycine is a donor of methylene groups, leucine and valine are nitrogen donors and metabolic fuel and serine is a donor of hydroxymethylene groups (77).

The cashew nut kernel amino acid profile in Table 5 is consistent with those from cashew nuts kernels from the geographically dispersed Vietnam, India, Brazil, and Ivory Coast (64, 78, 79).

## Nutrition and Phytonutrients and Their Potential as Functional Foods and Nutraceuticals

Plant foods contain many phytonutrients that have a positive effect on human health. Some of these are already used as or are being studied to understand their content in foods and if they can be used as functional foods or nutraceuticals. Functional foods are foods that exert a specific health benefit effect when they are consumed regularly (80). Nutraceuticals are food phytochemicals processed and made into pharmaceutical forms and dietary supplements, such as tablets, capsules, powders, and solutions, etc. (80). Phytochemicals from food that are available as nutraceuticals include anthocyanins, flavonols, hydroxycinnamate, and ellagic acid (80). Some foods from the Anacardiaceae family are used as or have the potential to be used as functional foods or nutraceuticals for their nutrient and phytonutrient content.

Dietary fiber is found in plants and includes carbohydrates and lignin with different properties and physiological effects. Plant fiber can beneficially affect health as functional foods through laxation and the delay of nutrient loss, attenuating blood glucose, normalizing serum cholesterol levels, reducing the risk of cardiovascular disease, may reduce breast cancer risk by altering sex hormone levels and may prevent colon cancer (81). Dietary fiber can enhance satiety and inhibit appetite which may enable it to be used in weight management to reduce calorie intake (81, 82).

Mango contains a relative abundance levels of phytonutrients in its pulp including lupeol, mangiferin and phenolic acids (53). Mango has anti-diabetic, anti-oxidant, anti-viral and anti-inflammatory properties as well as compounds with other health benefits (55). Mango peel and the flesh that attaches has the possibility of being used as a functional food (83) as it is a rich source of soluble dietary fiber (12.8–23.0%), insoluble dietary fiber (27.8–49.5%) and total dietary fiber (40.6–72.5%) and contains galactose, glucose and arabinose as well as bound polyphenols and flavonoids (84). It has a high water absorption capacity of 7 mL/g and swelling volume of 21 mL/g which could lead to its inclusion in food ingredients as a dietary fiber source and a functional food (85). These water absorption and swelling capacity are higher in mango peel fiber then from mango fiber concentrate made from combined peel and pulp which has a water holding capacity of 6.4 g/g, swelling capacity of 4.6 mL/g and oil holding capacity of 1.6 g/g (83).

Analysis of the effect of mango peel extracted with methanol on 3T3-L1 pre-adipocyte cell line show that some mango cultivars can inhibit adipogenesis, inhibiting mitotic clonal expansion formation of fat cells (adipocytes) and could potentially be a source of nutraceuticals to be used for obesity and to prevent an increase in fat mass (86). The gallotannin derivatives from mango can work in part through the AMO-activated protein kinase pathway to suppress adipogenesis in adipocytes (87). Clinical trials show that daily consumption of mango for 6 weeks can significantly increase systemic exposure to gallotannin-metabolites with implications for gallotannin-derived health benefits and gut microbial composition with body mass index associated differences of effect on them (88). Mango seed has antiplatelet aggregation effects with 72% inhibition that may be due to the mangiferin it contains (89). Mango peel phenolic compounds are able to be encapsulated and stabilized (90) which could lead to their use as a nutraceutical.

Clinical studies on the beneficial effects of cashew apple juice and the health benefits obtained by supplementation with it show a number of promising properties. Supplementation with cashew apple juice of men performing regular exercise gave lower carbohydrates and higher fat oxidation rates than those on a placebo (91). Daily consumption of the juice for 12 weeks gave an improved oxidative stress status shown by a decrease in malondialdehyde levels, an increase in plasma glutathione peroxidase and enhanced physical performance (92). There was enhanced physical performance shown by increased endurance and strength in cyclists who consumed the cashew apple juice for 4 weeks (93), and improvement in immunological mechanisms occurred as seen by increases in resting neutrophil counts and exercise-induced leukocyte counts (94). The high levels of vitamin C in the juice gives increased resting vitamin C levels in people who supplemented daily (91). Cashew nut consumption may be able to reduce systolic blood pressure (57).

Clinical trials of pistachio nuts suggest they may have a beneficial effect on the blood lipid profile and therefore cardiovascular health when they replace other calories, due to their unsaturated fatty acids, phytosterols, dietary fiber, protein, and magnesium (95). A 24 weeks study of patients with metabolic syndrome who ate pistachios as 20% of their total energy showed an improvement in lipids and a decrease in waist circumference (96). Patients with mild dyslipidemia who ate 40 g of pistachio's a day for 3 months had an increase in high-density lipoprotein cholesterol and decrease in low-density lipoprotein cholesterol, a decrease in total cholesterol and a decrease in fasting blood sugar (97). Obese mice with regular consumption of pistachio nuts have improved inflammation that could be related to positive modulation of the gut microbiota (79). Obese mice on a pistachio diet have had significant reduction in serum triglycerides and cholesterol and are able to reduce metabolic and cellular dysfunctions in the brain which may be useful in preventing obesity-related neurodegeneration (98). Diabetic mice who consumed a diet of pistachio nuts have increased gut populations of lactobacilli and bifidobacteria and normalized microbial flora was restored in them (99).

Pistachio nuts have anti-mutagenic potential and cytoprotective capacity (78). Pistachio kernel extracts have been shown to cause a significant decrease in cell viability and cell death of MCF-7 breast cancer cells (100). Pistachio nuts contain melatonin which has antioxidant capacity that can be protective against reactive oxygen and nitrogen species and sphingolipids which modulate cell health (101). The antioxidant capacity of pistachio nuts can be increased with the use of regulated deficit irrigation which may increase the production of secondary metabolites (78). Xylan has been isolated from pistachio nuts and can be used to produce a prebiotic mixture of the xylooligosaccharides xylobiose and xylotriose that may be suitable for functional or pharmaceutical use (102). Pistachio powder has been used to produce fortified bread that is enriched with lysine (103). Pistachio green hull contains a range of metabolites that benefit human health with antioxidant, photoprotective, cytoprotective, anti-inflammatory, anti-melanogenic, and anti-mutagenic activity (51).

The seeds of the Burdekin plum have been shown to have antihyperglycaemica and antihyperlipidemic effects on rats by significantly reducing the levels of blood glucose, total cholesterol, total triglycerides, and low density lipoprotein cholesterol. The seeds contain the phenolic compounds catechin, gallic acid, paramethoxybenzaldehyde, and pyrogallol (104). The fruit have also shown a cytotoxic effect against breast adenocarcinoma and laryngeal carcinoma human tumor cells and a moderate cytotoxic effect on human hepatoma cells (105).

## Allergens and Anti-Nutritional Compounds

The family Anacardiaceae contains plants that are known to cause allergic reactions (106). Approximately 32 genera in the Anacardiaceae family have been found to contain compounds that cause dermatitis upon contact (107). Plants in the Anacardiaceae family that are known to cause contact allergies include poison ivy (*Toxicodendron radicans*), poison oak (*Toxicodendron toxicarium*), poison sumac (*Toxicodendron vernix*), Chinese lacquer tree (*Toxicodendron vernicifluum*), African poison ivy (*Smodingium argutum*), cashew nut, pistachio nut, and mango (106, 108). Chironji has also been shown to have allergenic potential in both mice and humans (109).

The main polyphenol compounds that cause the allergies in this family are the 3-alkyl and 3-alkenyl catechols, particularly the C_15_-Catechols and C_17_-Catechols, which are sometimes known as urushiols, and the C_15_-Resorcinols and C_17_-Resorcinols (108).

Oxidation of these catechols turns them to highly reactive ortho-quinones which react with proteins in the skin to form antigens (108). The catechols that occur in the *Toxicodendron* genus are very strong contact allergens (108). The response appears to be an Immonoglobulin-E (IgE) mediated response after sensitization (110). The protein that causes chironji allergies leads to an increase in allergenic mediators, such as IgE, IgG1, and histamine levels and increased release of mast cell degranulation mediators (111).

Allergies to these plants and the foods they produce can be life threatening (106). The cashew nut shell liquid is used in the chemical products industry and is considered dangerous and cannot be handled with bare hands (21). It has been shown to be effective as a toxin and molluscicidal against golden apple snails (*Pomacea canaliculata*) which are a pest in Thailand that destroy rice crops (112).

The allergic reaction caused by mango includes skin irritation and erythematous lesions (113), pruritic erythema and periorvital edema (114) which can become severe anaphylactic reactions including urticaria, deep-tissue swelling, difficulty breathing, dyspnea, rhinorrhea, and cardiopulmonary symptoms (115, 116). The allergic reaction to these plants may be immediate or delayed and can appear up to 2 weeks after the contact (108, 116). The known allergen plants poison ivy and poison oak are also in the Anacardiaceae family and the clinical features of allergic dermatitis from these plants and mango are very similar (113, 117). Allergic rashes to mango have occurred in people with no previous exposure to it but who have been previously exposed to poison ivy or poison oak (117), and patients who have known allergies to these plants have also had mango dermatitis (113). Other cross-sensitivities between Anacardiaceae plants have also been detected (118).

Another anti-nutritional factor in the Anacardiaceae family is the contamination of pistachio nuts with aflatoxins (119). Fungal contamination of pistachios can occur in the field, during harvest, in post-harvest operations or in storage and the *Aspergillus* fungus produces aflatoxins in the nut (119). Aflatoxins can cause acute intoxication resulting in death (119) and contain carcinogenic compounds (120) and are among the most potent mutagenic substances known (121). Fungal contamination is prevented in the pistachio nut industry by controlling the moisture levels, insect activity, and rodent activity in the crop (121). Maximum legal limits of aflatoxin content are used to prevent contaminated pistachio nuts from entering the food supply (119).

There may be no known allergies to green plums at present, as they are currently only eaten by Indigenous populations in Australia where there are not many of the other known Anacardiaceae allergen plants growing. However, the fruit should be studied for the resorcinol and catechol compounds so that it is known if people already sensitized to these compounds could have an allergic reaction to green plums. This would particularly be the case if they were to be exported to countries where sensitization and allergies to poison ivy and poison oak are common.

## Nutritional Importance for Populations

Fruit and kernels of some of the Anacardiaceae family play major roles in nutrition in the world as they are major food crops. They are grown and distributed on enormous scales and provide valuable nutrients to many individuals and to many populations. Some of them are eaten by small isolated communities and others are grown and exported to cities and countries across the world. Regardless of how widely spread their availability, they affect the health of individuals who eat them, and in turn their availability plays a role in population health. Although they are not considered staple foods, they add significant diversification into the diet and therefore add essential components of nutrition. One way to estimate the impact they might have on population health is by looking at the economic impact they have, which shows their widespread availability and therefore the large number of people in whose health and nutrition they play a role. The economic growth of these foods is also a measure of nutritional importance. Production and trade occurs as a result and response to consumption and the level of consumption shows the important roles that these foods play in diet and nutrition. The Anacardiaceae family contains some species that are economically important including nuts, fruit, ornamentals, oils, resins, lacquers, and tannins (1). The foods with the highest level of economic importance and therefore the highest global impact on nutrition are the mango, the cashew nut and the pistachio nut.

Mangoes are grown in over 90 countries and are distributed throughout these countries and internationally to many more (122). Mango is the predominant tropical fruit produced globally with an estimated 39.1 million tons produced globally in 2018 which accounted for more than half of total global fruit production of major tropical fruits that year (123). The global market size of processed mango products in 2018 was estimated to be $16.55 billion USD (18). The consumption of mango continues to rise and it has been forecast that the global compound annual growth rate of processed mango products will increase by 6.4% from 2019 to 2025 (18).

The cashew nut is economically important around the world in many countries including Brazil, India, Nigeria, and Vietnam (19). It is cultivated in Vietnam, India, Guinea-Bissau, Ivory Coast, Tanzania, Brazil, Benen, and other parts of Central Africa and South East Asia (124). The global production of cashews for 2019/20 was 790,000 metric tons (MT) (kernel basis) with India producing 170,000–195,000 MT, the Ivory Coast 149,000 MT, Vietnam 82,000 MT, and Tanzania 53,000 MT (124).

Pistachio nuts are a major tree nut produced in the world. In the 2019/2020 production year 694,068 MT were produced with 331,538 MT from the United States, 205,000 MT from Iran, 85,000 MT from Turkey and 55,000 MT from Syria. The US exported about 200,000 MT and Iran about 125,000 MT. The biggest importers of pistachio nuts were China who imported 100,000 MT and the European Union who imported 95,000 MT (125).

The chironji nuts are economically important as they provides income to tribal people in north, west, and central India because of the high value placed on the seed kernel (35, 63). Chironji adds nutrition to the diet of tribal people in India who harvest it from the wild and eat it (35). Similarly, in Brazil the yellow mombin are harvested wild and then sold at local markets or frozen as a pulp and sold commercially throughout the country (33).

The marula fruit is eaten throughout Africa and is considered a good food-security resource providing food during the “hungry season” when grain stocks are low and other crops are not yet ready for harvesting (26, 29). The fruit are eaten fresh or cooked and used for juice which can be boiled to a syrup (26). If the seeds are clean, dry and completely ripe they can be stored for months without deterioration and used as emergency food caches (26). They can be used to flavor dishes or pounded into flour or used like a nut in baking (26). The edible kernel of the marula is eaten as a snack food by children in Niger and could be used as a food supplement by the larger population providing essential energy in the diet (29). For children in these communities who mostly eat millet and other grains the marula kernel plays an important role in bringing diversity to the diet and providing critical nutrients (29).

Many Australian Aboriginal communities commonly eat the green plum as food and it is a favorite with children (126, 127). Finding ways of value-adding to native foods or being able to use them as value-add ingredients may increase the economic returns for the community, and could increase the ecological cultural and social benefits (128). The native Australian fruit are nutritionally important and could add to the global food supply and be used for diet diversification providing valuable nutrients and micronutrients to the supply chain and giving better nutrition and health outcomes and help to combat dietary deficiencies.

## Future Studies of the Green Plum

As a food of the Anacardeaceae family, further studies on the green plum should be done as the family contains a number of important foods that provide nutrition to individuals and populations. The initial study of its nutritional properties was on underripe green plums which yielded promising results (7) but further analysis on ripe green plums should be done to confirm these. Proximate and metals, minerals and trace elements could show if the ripe green plum flesh has high levels moisture and carbohydrate, and if it is also a good source of potassium. Analysis of the fat content and fatty acid profile of the flesh and the kernel could tell if it is consistent with other foods of the Anacardiaceae family and if it also contains the high levels of fat and the 18:1 oleic acid fatty acid that are seen in this family, particularly in the kernels. The underripe green plums have high folate levels (7) and a further study of the folate in the fruit as it matures and ripens could give valuable information about folate in fruit, particularly in this family, and as a comparison to the folate levels seen in the small underripe mangos (3). These nutritional studies could enhance the role that green plum already plays in providing nutrients to the Aboriginal communities of northern Australia and the potential role they could play in providing diet diversification to the larger population of Australia or globally, as other economically important fruit and kernels in this family are already doing.

Studies on the mango, cashew apple, pistachio nut and Burdekin plum show that they have potential as functional foods or nutraceuticals due to their nutritional content and the compounds they contain, thus, the green plum should be characterized to demonstrate if it has similar properties and potential for future use. The anti-nutritional properties in the family and particularly the allergy causing compounds found in some of the plants and fruit indicate that the green plum should be analyzed to find out if it also contains these compounds and if sensitization can occur from it. If storage trials indicated that fungal contamination could be a problem for green plums then aflatoxin and other toxin assays should be done to understand their contamination and risk.

## Conclusion

The fruit of the Anacardiaceae family provide many individual health benefits from the nutrients they contain. Some of them are major food crops that are grown and distributed across the world, providing essential nutrition to many populations. The fruit are good sources of carbohydrates, potassium and folate. Of some concern are the allergenic compounds that many Anacardiaceae fruit contain, and testing for these could be beneficial. The Anacardiaceae family has a number of nutritionally important kernels, providing high levels of protein, fat, potassium, phosphorous, and amino acids. The kernels are all high in oleic acid, linoleic acid, palmitic acid, and stearic acid. The green plum is a very small fruit and the kernel in the seed is very small, however, it could be of interest to study the green plum kernel to see if it also contains the valuable nutrients seen in these other kernels. Some Anacardiaceae fruit are being used as functional foods and if the green plum industry were to grow it could be of interest to understand the health functions they can play on the human body. As an Anacardiaceae fruit, the green plum has potential as a source of nutrition and diet diversification and further studies on it as a food are justified.

## Author Contributions

SF, HES, HJS, and YS planned the paper. SF researched and wrote the manuscript. SF, HES, HJS, MR, and YS edited the manuscript. HES, HJS, MR, and YS supervise the project and Ph.D. All authors contributed to the article and approved the submitted version.

## Conflict of Interest

The authors declare that the research was conducted in the absence of any commercial or financial relationships that could be construed as a potential conflict of interest.
